# Role of calcium-sensor proteins in cell membrane repair

**DOI:** 10.1042/BSR20220765

**Published:** 2023-02-27

**Authors:** ZiWei Li, Gary S. Shaw

**Affiliations:** Department of Biochemistry The University of Western Ontario, London, Ontario N6A 5C1, Canada

**Keywords:** calcium signalling, membrane fusion, trafficking

## Abstract

Cell membrane repair is a critical process used to maintain cell integrity and survival from potentially lethal chemical, and mechanical membrane injury. Rapid increases in local calcium levels due to a membrane rupture have been widely accepted as a trigger for multiple membrane-resealing models that utilize exocytosis, endocytosis, patching, and shedding mechanisms. Calcium-sensor proteins, such as synaptotagmins (Syt), dysferlin, S100 proteins, and annexins, have all been identified to regulate, or participate in, multiple modes of membrane repair. Dysfunction of membrane repair from inefficiencies or genetic alterations in these proteins contributes to diseases such as muscular dystrophy (MD) and heart disease. The present review covers the role of some of the key calcium-sensor proteins and their involvement in membrane repair.

## Introduction

The plasma membrane is a protective barrier for cells, made up of a phospholipid bilayer with embedded proteins that carry out specific functions. An intact plasma membrane is vital for cell integrity and maintaining homeostasis providing a stable, yet semipermeable, platform for proteins and lipids to conduct nutrient intake, vesical transport, enzyme secretion and signaling within and outside of the cell. Unlike cell walls in plant and bacterial cells that provide greater structural support and strength, the plasma membranes of animals are more fragile and susceptible to injuries from the surrounding environment that can damage the phospholipid bilayer, eventually leading to cell death. Multiple mechanisms are possible to tear or rupture the plasma membrane including injury from mechanical, chemical, or bacterial/viral sources [1,2]. For example, in cardiac and skeletal muscle, the high mechanical stress placed on muscle fibers due to repeated contractions causes the plasma membrane in these cells to frequently rupture. Over time, the constant breaching of the plasma membrane in cardiac muscle cells can lead to heart failure. Reactive oxygen species through mitochondrial dysfunction can cause oxidation of proteins and lipids leading to pores in cell membranes, especially in cells with large numbers of mitochondria and increased metabolic activity such as liver and brain [3–5]. Chemical and mechanical cell damage occurs in oral cavities due to brushing, chewing, and tobacco usage [6,7]. Damage to skin cells is induced by ultraviolet radiation and physical abrasion [8]. Defects in neuronal membrane integrity are common in neurological conditions, including traumatic brain and spinal cord injuries, Parkinson’s disease [9], and Alzheimer’s disease [10]. Myocardial damage including ischemia-reperfusion injury can lead to heart failure [11–13]. Infection by pathogenic bacteria, viruses, and pore-forming toxins (PFTs) can result in transmembrane lesions at the cell membrane [14,15]. Bacteria associated with Shigella or Salmonella that cause intestinal infections utilize a ‘needle-like’ protein apparatus to breach a membrane and secrete proteins into host cells [16]. The spike protein of SARS-CoV2 latches on to cells and then uses the host’s enzymatic system to fuse with the plasma membrane to create a pore that facillitates infection [17]. In these cases, it is unclear what damage to the plasma membrane has been left behind. Several excellent reviews on the mechanisms and outcomes of plasma membrane rupture are available [11,12,18,19].

Nonetheless, cells have a remarkable ability to survive membrane disruption, frequently involving complex networks of proteins and lipids that patch a membrane lesion and return the cell to normal function. Multiple studies have demonstrated that calcium is a necessary requirement for membrane repair following disruption. For example, membrane-fusion that seals a torn plasma membrane is observed in sea urchin eggs, triggered by the rapid influx of extracellular calcium ions (Ca^2+^) [20]. In contrast, membrane damage is not repaired when Ca^2+^ is absent from the external environment, resulting in continuous spillage of cytoplasmic contents and cell death. Furthermore, upon microelectrode puncture wounding of frog skeletal muscle fibers seal effectively in the presence of Ca^2+^ but is suppressed at low Ca^2+^ concentrations [19]. In addition, frog oocytes wounded by microneedle fail to reorganize microtubules and heal the lesion in a Ca^2+^-free environment [21]. The key step in these membrane repair processes is the sensing of abnormal cytosolic Ca^2+^ concentrations by specific membrane repair proteins including dysferlin, synaptotagmins (Syt), caveolins, TRIM72, annexins, and others. Several excellent reviews have been published that describe different aspects of membrane repair [22–27]. In the present review, we focus on the roles of calcium-sensor proteins in membrane repair and the implications of dysfunctional membrane repair proteins on human disease.

## Defective membrane repair is causative for multiple human diseases

**Figure 1:**
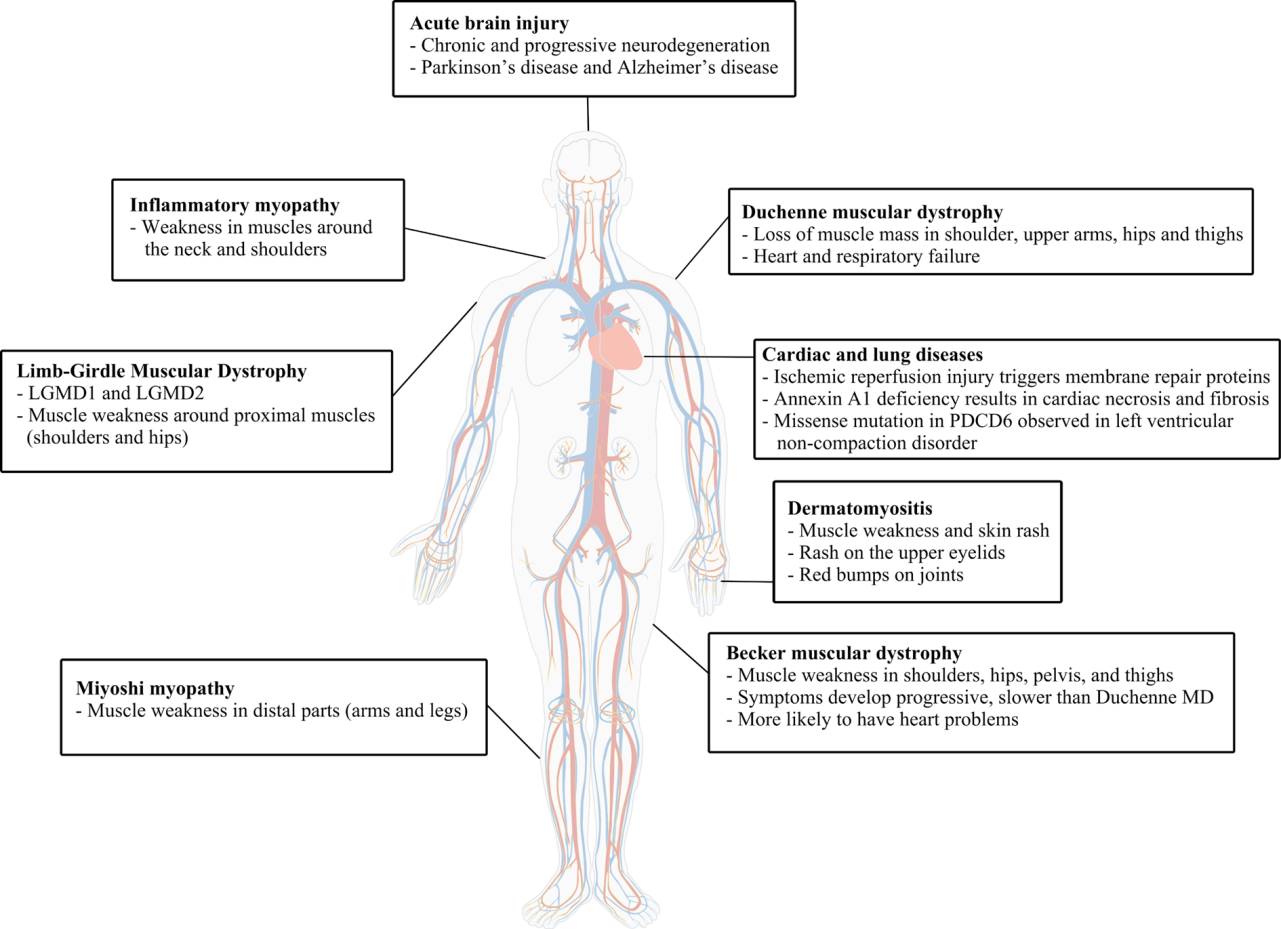
Pathological conditions and symptoms associated with membrane repair pathways Duchenne and Becker MD result from loss of muscle mass. Duchenne MD is also associated with increased risk of heart and respiratory failure, while Becker MD patients develop symptoms slower than Duchenne MD. LGMD (LGMD1 and LGMD2) manifests in the proximal muscles around the hips and shoulders. Miyoshi myopathy manifests in distal muscles, mainly in legs. Inflammatory myopathy is associated with weakness in the large muscles around the neck, shoulders, and hips. Two types of inflammatory myopathy are polymyositis and dermatomyositis that cause lung inflammation. Acute brain injury can lead to chronic and progressive neurodegeneration such as Parkinson’s disease and Alzheimer’s disease. Defects in membrane repair contribute to different cardiac and lung diseases.

Pathological conditions and chemical exposure that compromise the membrane repair process or membrane integrity pose immediate threats to cell survival. Generally, the location of the defective repair process dictates the type of illness or disease. Mutations in several genes that encode membrane repair proteins contribute to diseases such as muscular dystrophy (MD), gastrointestinal diseases, and heart disease [28,29]. The development and maintenance of skeletal and cardiac muscle is particularly affected (Figure 1) [30]. For instance, according to the Human Gene Mutation Database (www.hgmd.cf.ac.uk), nearly 400 missense mutations in the *DYSF* gene, responsible for the membrane repair protein dysferlin, are documented to cause weakness and deterioration in muscles of the arms and legs and manifested in multiple forms of limb-grindle muscular dystrophy (LGMD), Miyoshi myopathy, or general dysferlinopathies. Patients with Miyoshi myopathy also display early myocardial dysfunction prior to developing more serious cardiovascular problems. Similarly, over 350 mutations in the *CAPN3* gene (calpain-3) have been identified where >90% are linked to LGMD. Mutations in the transmembrane protein caveolin-3, also involved in membrane repair, are causative for LGMD and ‘rippling-muscle’ disease but also implicated in cardiomyopathies and sudden-infant death syndrome [31]. Some rare disorders such as chronic inflammation of muscle cells are also characterized by changes in proteins involved in membrane repair [32]. An uncontrolled autoimmune response in skeletal muscle is believed to begin with a deficiency of Syt VII (Syt7) that results in autoantibodies-targeting TRIM72. The continued inflammatory response eventually leads to muscle degeneration [30,33,34]. While not involved in membrane repair dystrophin co-ordinates a large protein complex near the sarcolemma of muscle cells to maintain membrane integrity and prevent injury, especially during contraction. Deletions, truncations, or mutations of the gene (DMD) for dystrophin are responsible for Duchenne or Becker forms of MD [30,35,36].

Multiple studies show that defects in membrane repair are manifested in different cardiac and lung diseases. The oxidative stress of ischemic injury and subsequent reperfusion is thought to trigger membrane repair proteins. Dysferlin, annexins, and TRIM72 are up-regulated in the latter stages of heart failure [11,37], perhaps in a last-ditch effort to rescue cardiac cells. Annexin A1 (ANX1) deficiency results in cardiac necrosis and fibrosis following myocardial infarction and other annexin proteins are up-regulated in a failing heart [38,39]. Recombinant TRIM72 has been shown to have a protective role and stabilize myofibers [40] and improve membrane repair in the heart [41,42]. As a result, administration of TRIM72 has been suggested as a possible therapeutic route following myocardial infarction [43]. Truncation and missense mutations have been observed in the repair protein AHNAK in congenital heart disease patients [44] and a missense mutation in apoptosis-linked gene 2 (ALG-2, also called programmed cell death 6 protein [PDCD6]) is observed in patients with left ventricular noncompaction [45]. Impaired left ventricle function has also been identified in patients carrying missense mutations in the dysferlin gene. In the lungs, both caveolin-1 and TRIM72 are important in the repair of epithelial cells in lung tissue [46,47]. The absence of either of these proteins causes abnormalities and diminishes the repair process.

Currently permanent solutions for any form of MD or inflammatory myopathies are not available for patients [11,30]. However, stimulating membrane sealing with gene therapy approaches appears to be a promising therapeutic strategy. Gene replacement strategies using overlapping viral vectors have been used in mice [48]. Exon skipping has been used to replace protein-coding and noncoding regions in the *DYSF* gene to rescue membrane repair in patient fibroblast cells [49]. In addition, CRISPR methods in patient-derived stem cells can be used to replace missense mutations in the *DYSF* gene [50].

## A calcium surge triggers membrane repair

The Ca^2+^ ion is accepted as an evolutionarily conserved cellular signaling molecule originally shown to control heart contraction and neurotransmitter release [54,55]. Through binding to a wide variety of proteins, Ca^2+^ has the ability to initiate conformational changes that signal muscle contraction, synaptic transmissions, and enzymic function [56]. Furthermore, Ca^2+^ can neutralize the negative potential of proteins and membrane surfaces fine-tuning electrostatic gradients in the cell. The observation that nearly all aspects of cellular life are affected by Ca^2+^ requires intracellular Ca^2+^ concentrations to be tightly regulated. Cytosolic Ca^2+^ concentrations are maintained near ∼100 nM by calcium transporters that actively pump Ca^2+^ ions from the cytosol to extracellular space (Na^+^/Ca^2+^ exchangers, voltage-dependent Ca^2+^ channels), from the cytosol to the endoplasmic reticulum (sarco/endoplasmic reticulum calcium ATPase, SERCA) or Ca^2+^-channels (ryanodine receptor, IP3 receptor) that release calcium from the endoplasmic reticulum to the cytosol [56,57]. In addition, calcium-sensor proteins with high affinities (*K*_d_ = 10^−9^ M) or high calcium capacity act as sponges to limit Ca^2+^ in the cytosol [58]. This results in steep concentration gradients between cytosolic Ca^2+^ levels and extracellular (2–3 mM) or intracellular storage (0.5–1 mM) environments. In many signaling processes, intracellular Ca^2+^ concentrations increase to 1–10 µM, controlled by Na^+^/Ca^2+^ exchangers or voltage-gated Ca^2+^ channels, in response to ligand binding or changes in membrane potential. Here, EF-hand Ca^2+^-binding proteins such as troponin-C and calmodulin have calcium affinities that are finely tuned to bind Ca^2+^ ions, undergo conformational changes and modulate interactions with other proteins to stimulate muscle contraction or enzymatic action [59–62].

Unlike this controlled import of Ca^2+^ in response to stimuli, membrane rupture causes a rapid, unrestrained influx of extracellular Ca^2+^ into a cell around the rupture site, resulting in an overwhelming surge in cytosolic Ca^2+^ [63]. In the absence of appropriate mechanisms to reseal the membrane fault, toxic levels of intracellular Ca^2+^ promote apoptosis and cell death. For small pores (<1 nm), membrane repair likely occurs using thermal fluctuations of phospholipids to reseal a hole, first observed in liposomes and red blood cells [64,65]. The mechanism relies on the unfavorable lipid disorder energetics of the hydrophobic phospholipid backbone exposed to the hydrophilic surroundings created at the rupture site [64,66]. As a result, an increase in surface energy drives lipids at the rupture site to form curved edges to minimize pore edge tension. Most injuries induced by mechanical or chemical stress produce larger membrane holes (>10 µm^2^) that are repaired rapidly (<10–30 s) [67,68]. In the presence of additional oxidative stress, this time frame can be extended (100–300 s) [69]. In all cases, membrane lesions require specialized repair mechanisms to detect and determine the nature of the injury, then treat it with Ca^2+^-sensitive proteomic machinery.

## Membrane-resealing pathways

**Figure 2:**
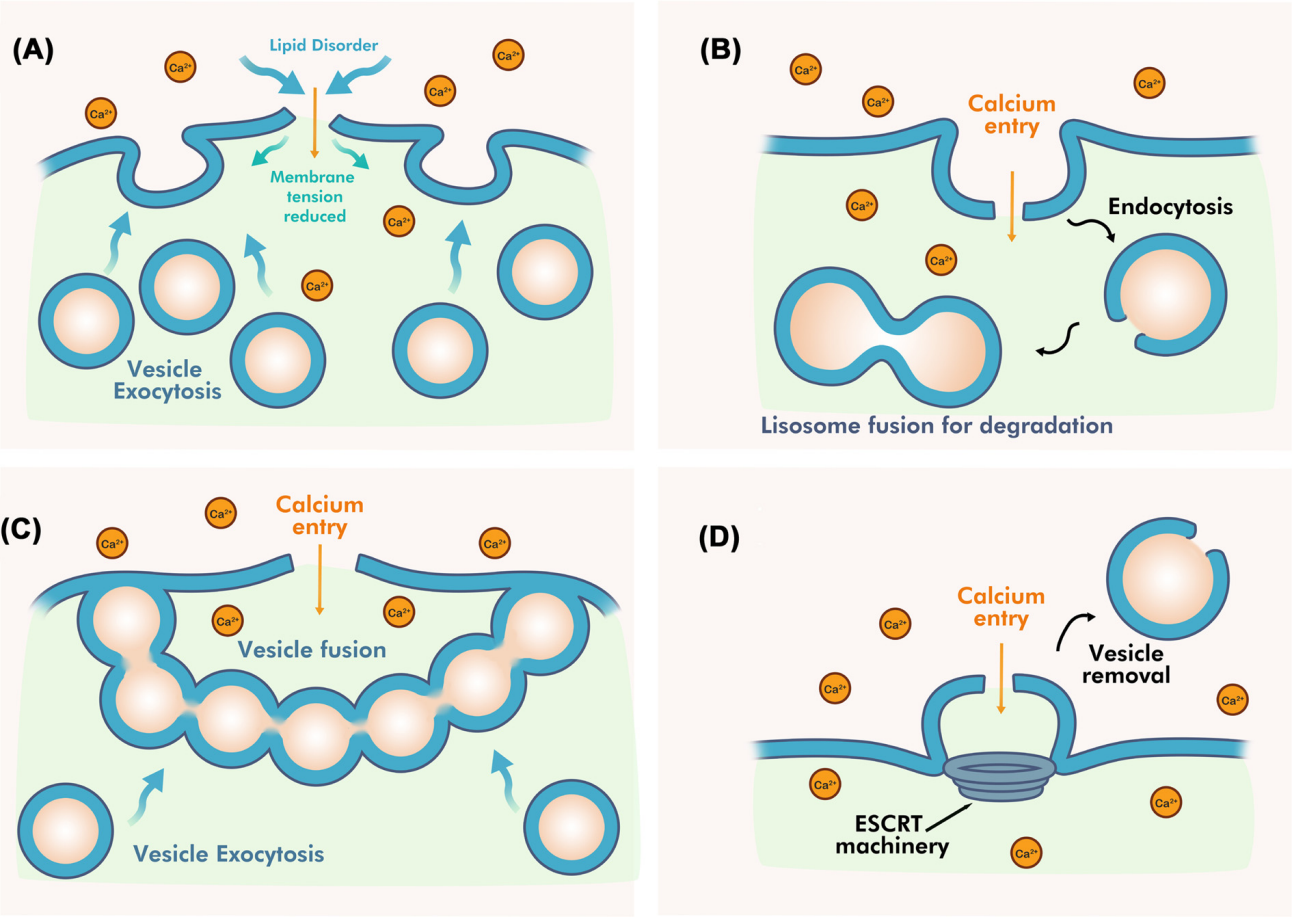
Active membrane repair pathways Larger lesions in a plasma membrane are repaired by (**A**) Exocytosis, (**B**) Endocytosis, (**C**) Patching and (**D**) Shedding. (**A**) Calcium influx activates vesicular exocytosis events. (**B**) Inward budding reseals small lesions (<100 nm) by creating lesion-containing vesicles that fuse with lysosomes targeted for endo-lysosomal degradation. (**C**) Exocytotic-induced vesicles fuse at the rupture site, forming a large patch that isolates the rupture site. (**D**) Shedding repairs holes by outward budding and pinching off of vesicles containing the damaged portion of the plasma membrane.

In general, there are about four calcium-sensitive mechanisms used by cells to repair larger holes in the plasma membrane: exocytosis, endocytosis (internalization), patching, and shedding (externalization) (Figure 2) [20,26,70–72]. In exocytosis, damage to the plasma membrane triggers Ca^2+^ influx and the localization of intracellular vesicles to the damaged site within seconds. Subsequent fusion of vesicles to the inner surface of the plasma membrane has been observed in sea urchin eggs, endothelial cells, and fibroblasts (Figure 2A) [20,73]. This mechanism that artificially adds more lipids via vesicle fusion to a broken plasma membrane reduces membrane tension efficiently. Membrane patching is similar to the exocytosis-mediated repair mechanism and also utilizes intracellular compartments. This Ca^2+^-dependent vesicle–vesicle fusion event is observed in starfish oocytes and sea urchin eggs [67]. The patching model relies on the rapid Ca^2+^-dependent fusion of vesicles to form a large multicompartment barrier that encompasses and fuses with the plasma membrane at the rupture site. This model allows sealing of much larger holes in the membrane (>10 nm in diameter) within seconds. The underlying proteomic machinery for exocytosis and patching requires Ca^2+^-sensor proteins that detect the injury sites and promote vesicle–vesicle and vesicle–plasma fusion events. Candidate Ca^2+^ sensor proteins for these mechanisms include members of the Syt and annexin protein families and dysferlin [12,74–77].

The endocytosis mechanism reseals membrane holes (<100 nm) through inward budding where the damaged plasma membrane is pinched off. The resulting lesion-containing vesicle then fuses with lysosomes and is targeted for endo-lysosomal degradation [78]. Membrane lesions induced by the bacterial toxin streptolysin O (SLO) show rapid Ca^2+^-dependent resealing by this mechanism [79]. The shedding mechanism repairs holes by outward budding of vesicles containing the damaged portion of the plasma membrane [80]. This mechanism utilizes proteins of the **E**ndosomal **S**orting **C**omplex **R**equired for **T**ransport (ESCRT) machinery to repair smaller holes (<100 nm) [71]. As with endocytosis, the shedding mechanism appears to be used to repair SLO-infected cells [72,79]. In addition, Ca^2+^ activation promotes the accumulation of multiple proteins near the site of the membrane lesion, assembling a barrier that plugs the lesion to limit the loss of cytosolic media. The annexin protein family are major Ca^2+^ sensors for this protein accumulation process [81], and annexins appear to work with other proteins including the S100 proteins, dysferlin, and TRIM72 (MG53) during membrane repair [82–84].

## Different roles for Ca^2+^ sensor proteins in membrane repair

### Syt1 and Syt7 and soluble *N*-ethylmaleimide-sensitive factor attachment protein receptor proteins in exocytosis

The Syt family of Ca^2+^-binding proteins regulates Ca^2+^-dependent exocytosis in several cell types. The process is especially well characterized in neurons for neurotransmitter release from synaptic vesicles [85]. Syt proteins consist of 17 isoforms in mammals that each have an N-terminal transmembrane region, a variable length linker region and two cytosolic C2 domains (C2A, C2B) [86–88], first identified in protein kinase C [89,90]. Multiple structures of C2 domains from different Syt proteins show these share a conserved β-sandwich fold of eight antiparallel β-sheets connected by surface loops [91–96]. Most isoforms show tissue-specific distribution, whereas only nine isoforms (Syt1–Syt7, Syt9, Syt10) have C2 domains that bind calcium [97]. Typically, C2 domains bind two to three Ca^2+^ ions although up to four sites are identified when comparing multiple family members [98–100]. Calcium binding is controlled by a series of loops and water molecules at one end of the β-sandwich structure. The C2A domain of Syt1 binds three Ca^2+^ ions with *K*_d_ of 54, 530 μM, and a very low affinity of >20 mM. Its C2B domain binds two Ca^2+^ ions with *K*_d_ of 300–400 and 500–600 μM [100–102]. However, the intrinsic Ca^2+^ affinities of the Syt proteins are distinct in different isoforms. For instance, Syt7 has the highest Ca^2+^ sensitivity, having a half-maximum binding of Ca^2+^ at 1.5 μM for the C2A domain and 2.5 μM for the C2B domain [103]. Ca^2+^-binding does not cause a significant conformational change in the C2 domains, rather it modifies the electrostatic surface potential that allows the C2 domains to interact with phospholipids and intensifies Ca^2+^ binding [91,99,104]. As a result, substitutions to ligating residues in Syt severely dampens both Ca^2+^ binding and phospholipid interactions.

**Figure 3:**
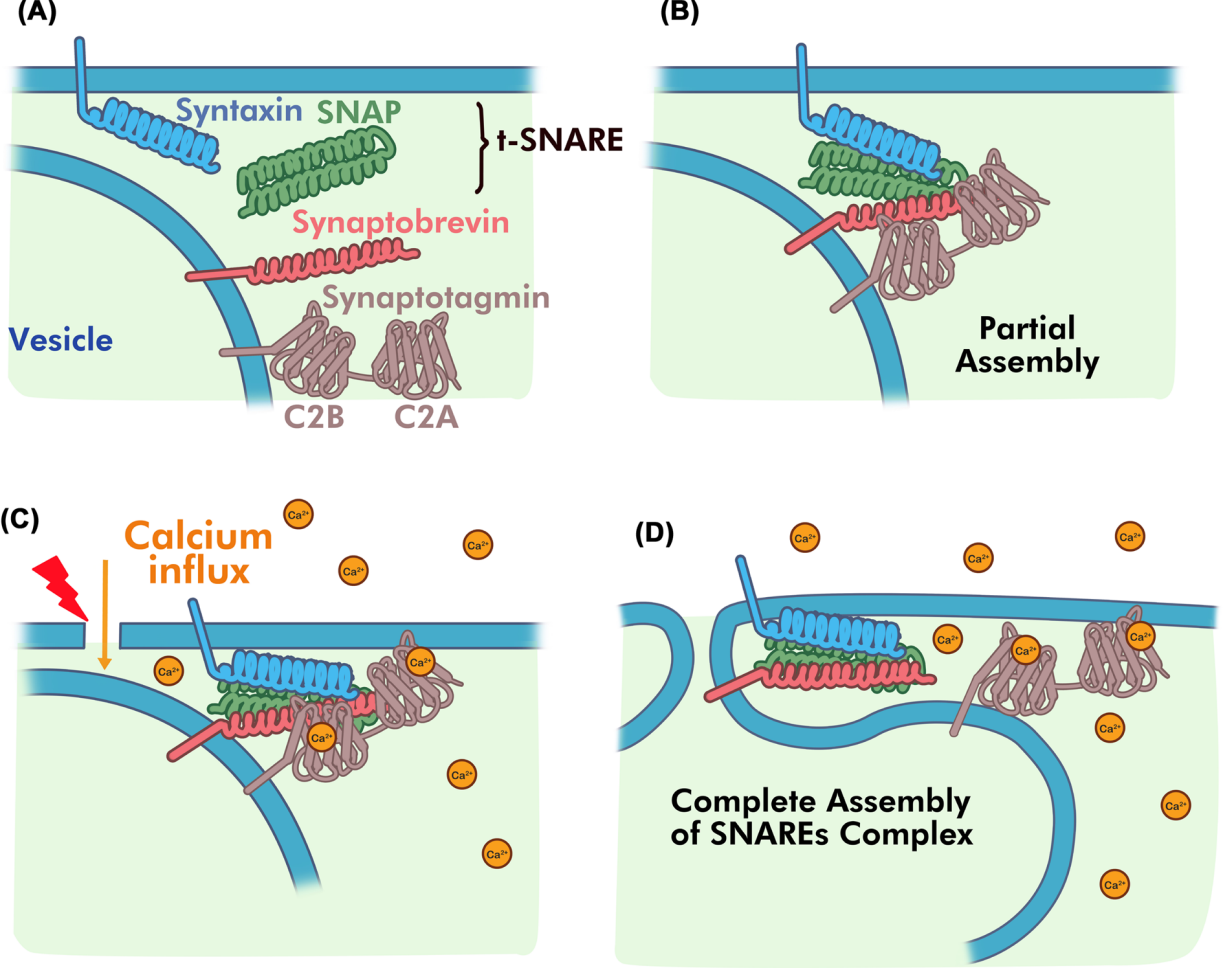
Model of SNARE assembly and membrane fusion (**A**) Locations of the SNARE and Syt1 near the plasma membrane vesicle membrane interface in the absence of calcium influx. The SNARE proteins syntaxin1 (cyan) and SNAP-25 (green) localize on the plasma membrane while synaptobrevin 2 (red) and Syt1 (gray) localize to the vesicle membrane. The C2A and C2B domains of Syt1 are not bound to Ca^2+^. (**B**) In the prefusion state, Syt1 interacts with the partially assembled SNAREs to form an inhibitory complex in a Ca^2+^-independent manner. (**C**) Ca^2+^ channel opening causes Ca^2+^ influx, resulting in Ca^2+^ binding to the Syt1 C2 domains. (**D**) The Ca^2+^-bound C2 domains intercalate into the neighboring plasma or vesicle phospholipid membrane releasing the SNARE complex.

The most comprehensively studied Syt isoform is the neuronal Syt1, shown to be the principal Ca^2+^ sensor that triggers neurotransmitter release through exocytosis [105,106]. Syt1 functions with the Soluble *N*-ethylmaleimide-sensitive factor Attachment protein Receptor (SNARE) proteins synaptobrevin 2, SNAP-25 (25 kDa synaptosomal-associated protein) and syntaxin 1 to trigger membrane fusion between a synaptic vesicle and the plasma membrane [107]. SNARE proteins are anchored to the vesicle (synaptobrevin 2) or plasma (SNAP-25, syntaxin 1) membrane via either a transmembrane domain or post-translation modification (Figure 3A). Although multiple models have been proposed [108–111], one recent model based on X-ray structures indicates assembly of the SNARE proteins into a partial four-helix bundle structure may be important to understand the exocytosis model for the repair progress [112,113]. This remains to be validated.

Evidence suggests that in the absence of Ca^2+^, a prefusion complex is formed between the three SNARE proteins, the cytosolic protein complexin and Syt1 that brings the vesicle and plasma membrane surfaces into close proximity (<4 nm, Figure 3B) [112,114,115]. This allows the SNARE proteins to form a partial four-helix bundle in complex with two molecules of Syt1 that reside on a synaptic vesicle. One molecule of Syt1 interacts with the syntaxin/synaptobrevin/complexin components while a second molecule interacts on the opposite side of the SNARE complex with SNAP-25. In both cases, the C2B domain appears to be the major interacting region with the SNARE complex. Upon calcium entry, and Ca^2+^ binding to Syt1 (Figure 3C), the Syt1 interaction with synaptobrevin/complexin is released and the Ca^2+^-filled C2 domains intercalate with either the plasma or vesicle membranes that allows the SNARE proteins to ‘fully-zipper’ and facilitate fusion of the two membranes (Figure 3D) [111]. The sensing role of Syt1 is critical for full SNARE assembly and release of inhibition since deletion of Syt1 or substitutions in the C2B domain impair neurotransmitter release [116]. The energy released from the full assembly of the SNAREs, and neutralization of the acidic Ca^2+^-binding loops in Syt1 appear to be the driving force to overcome fusion of the similarly negative-charged vesicle and plasma membranes [115,117,118]. Loss of Syt1 causes a reduction in membrane integrity that is magnified under stress conditions such as salt and freezing in plants [119,120].

Syt7, widely expressed in neuronal and non-neuronal tissues, is proposed to have a calcium-sensing role in membrane repair. Syt7 is located on the membrane surfaces of lysosomes and nonsynaptic secretory granules. Early work showed that Syt7 regulates membrane repair in fibroblasts via calcium-sensitive lysosome exocytosis [121]. Knockdown of Syt7 results in a reduction in exocytosis of pancreatic β-cells’ secretory granules [122] and defects in lysosomal exocytosis and membrane repair are noted in Syt7-deficient mice [34]. Syt7 shares some features with Syt1 and mechanistic features for possible membrane repair by Syt7 can be gleaned from the role of Syt1 in synaptic vesicle fusion. For example, *in vitro* assays show Ca^2+^ binding promotes SNARE-mediated membrane fusion. Syt7 exhibits strong Ca^2+^-sensitive binding to phospholipids and the plasma membrane SNARE proteins syntaxin 1A and SNAP-25 [123]. Furthermore, lysosomal exocytosis studies show interactions between Syt7 and SNAP-23 are facilitated by syntaxin 4, but not by syntaxins 2, 3, or 6 [124]. Pulldown assays detect interactions between TI-VAMP/VAMP7 and SNAP-23 with the C2A domain of Syt7 under 1 mM Ca^2+^ but not with the Syt1 C2A domain [124]. Botulinum neurotoxin E cleavage of SNAP-23 severely inhibits lysosomal exocytosis, indicating that interactions of Syt7 with some isoforms of SNARE proteins may play a key role in membrane resealing [124].

### Membrane patching by dysferlin

The membrane repair protein dysferlin is a large, tail-anchored transmembrane protein in the Ferlin family of vesicle fusion proteins. It is expressed in many tissues [76] but is particularly abundant in skeletal and cardiac muscle where dysferlin is localized to cytoplasmic vesicles and the sarcolemma. Dysferlin-deficient mice display defective Ca^2+^-dependent sarcolemma repair and develop forms of slow, progressive MD [125–127]. Consistent with its role in membrane repair, mutations in the dysferlin gene are linked to two diseases characterized by muscle weakness, Miyoshi myopathy and LGMD type 2B (LGMD2B) [51,52]. Furthermore, immunolabeling experiments of dystrophic skeletal muscle display extreme reductions of dysferlin at the sarcolemma [128]. Dysferlin-null myofibers initially survive contraction-induced injury but eventually succumb to inflammation and necrosis in the absence of membrane repair. On the other hand, wild-type muscle cells can survive large-strain injury by sarcolemma membrane repair [126]. Reintroduction of the dysferlin gene into dysferlin-null mice promotes rapid recovery of skeletal muscles from contraction-induced injury [129]. Dysferlin-deficient cells are more prone to injury following mechanical membrane stress [130,131]. These findings suggest that dysferlin is vital in mediating the recovery of membrane rupture and maintaining membrane integrity in muscle fiber cells.

Dysferlin comprises seven C2 Ca^2+^-binding domains (C2A–C2G) and two accessory domains (C2-FerA, DysF) anchored to vesicle surfaces and the sarcolemma in muscle cells by a C-terminal transmembrane domain. *In vitro* studies show the C2 domains bind one to two Ca^2+^ ions and display a wide range of Ca^2+^-binding affinities [132]. For instance, dissociation constants (*K*_d_) between 1 and 3 μM have been measured for individual sites in the C2A and C2C domains, whereas sites in the C2D and C2E domains have affinities well above 500 μM [132]. Protein–lipid overlay assays show that the dysferlin C2 domains have diverse affinities and Ca^2+^ dependencies for different lipid types. All C2 domains of dysferlin bind phosphatidylserine (PS) lipids in a Ca^2+^-independent fashion that is enhanced in four of the C2 domains (C2A, C2E, C2F, and C2G) upon Ca^2+^ binding [132]. The C2A domain exhibits the highest Ca^2+^-induced association with PS and phosphoinositide [133]. High-resolution structures are available for the C2A domain [134,135] and the DysF domain [136,137] from dysferlin. Like the Syt proteins, structures show that the C2A domain forms a β-sandwich structure with the Ca^2+^ loops at one end of the fold [134,135]. Missense mutations in each of the C2 domains of the dysferlin gene cause rare forms of MD, implying that altering a single C2 domain is sufficient to disrupt its membrane repair function [138]. This could arise from defects in domain folding, association with binding partners, improper lipid interactions or alterations in the overall tertiary structure [138,139]. Ca^2+^ binding to the C2A domain does not induce large conformational changes but rigidifies the Ca^2+^-binding loops important for lipid binding [135]. Structures of the remaining C2 domains are not available but have been predicted by RoseTTAFold and AlphaFold2 programs. Early predictions suggested that dysferlin adopts a flexible ‘beads on a string’ tertiary structure with the C2A domain furthest from the membrane surface [140,141]. More recently, modeling algorithms predict a more compact arrangement where all the C2 domains are packed together, along with the C2-FerA and DysF domains [142].

**Figure 4:**
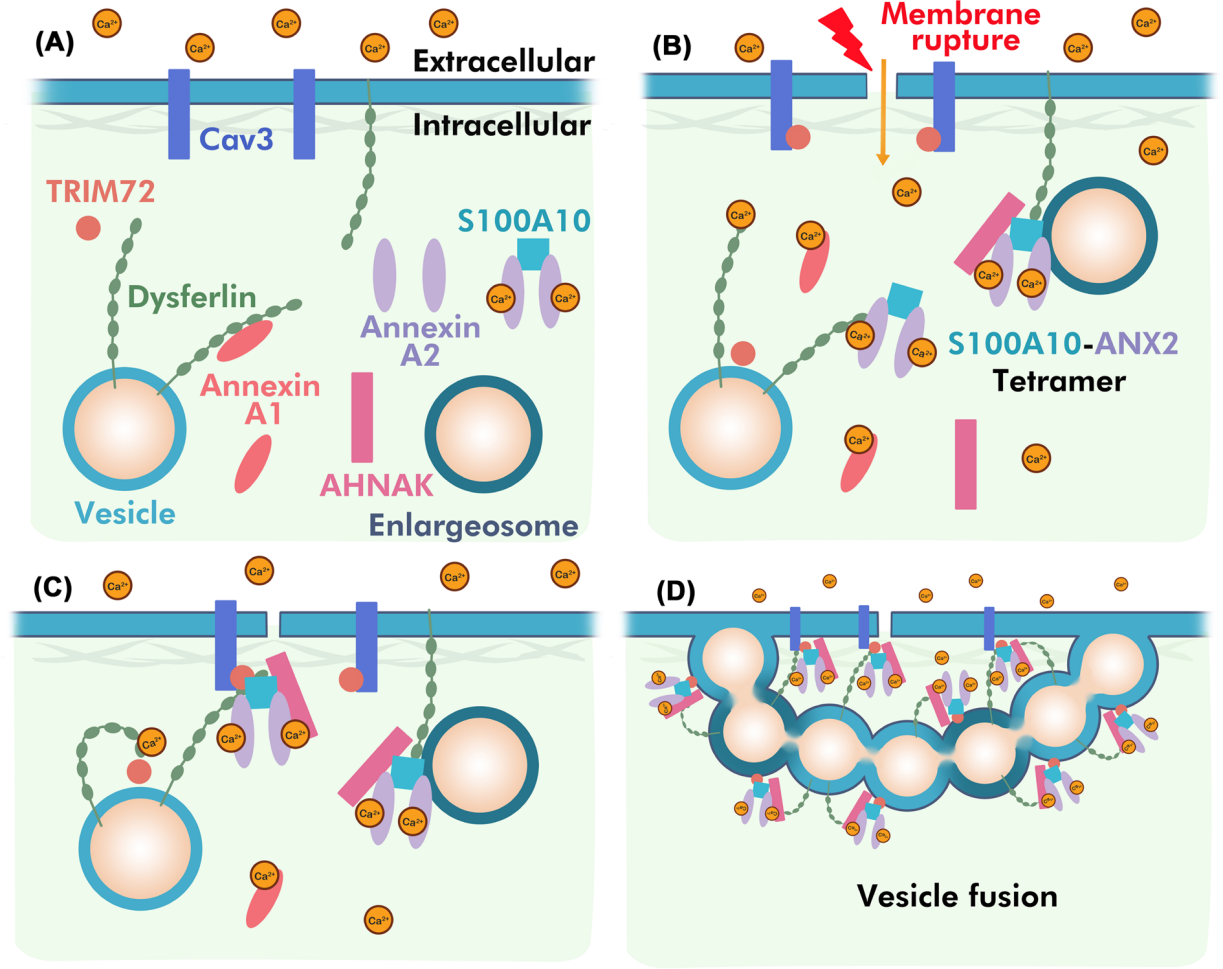
Model of dysferlin-mediated vesicle fusion membrane repair (**A**) Dysferlin associates with plasma and vesicle membranes. Other proteins including TRIM72, AHNAK, and S100A10:ANX2 are mostly cytosolic. (**B**) Membrane rupture causes a local Ca^2+^ influx, binding to and activating Ca^2+^-sensitive proteins such as dysferlin and ANX2. This enables multiple interactions between the proteins and causes recruitment of TRIM72 and AHNAK by dysferlin and S100A10:ANX2 to vesicle surfaces. (**C**) Ca^2+^-dependent protein–protein interactions recruit vesicles such as enlargosomes to the injured plasma membrane facilitated by the complete dysferlin complex that spans the vesicle-plasma membrane space. (**D**) Multiple vesicles containing dysferlin and interacting proteins accumulate at the injured site forming a patch across the membrane lesion.

Dysferlin is proposed to direct calcium-sensitive membrane patching of the sarcolemma in skeletal and cardiac muscle cells (Figure 2C) [143]. This process is triggered by a rapid Ca^2+^influx into cells causing a high Ca^2+^-concentration zone around the rupture site. Vesicles recruited to the injury site fuse with one another forming a patch that anneals with the plasma membrane to reseal the rupture sites (Figure 4). Live-cell imaging using mechanically wounded rat myocytes shows dysferlin-containing vesicles transport along microtubules toward the injury site [144]. It is unclear what vesicles are involved, yet these undergo rapid fusion to form larger vesicles in the cytoplasm rather than directly accumulating at the rupture sites. The proposed role of Ca^2+^ is to trigger formation of large dysferlin-containing vesicles that act as a ‘patch’ to repair large lesions [144]. Since dysferlin contains a transmembrane domain anchored to phospholipids in both vesicle and plasma membranes, it is thought to co-ordinate vesicle docking and fusion with help from cytosolic protein-binding partners that do not contain membrane-spanning regions such as S100A10, Annexin A2 (ANX2), AHNAK, and TRIM72 (MG53) [82,145,146]. Supporting this, multiple large-scale proteomics experiments have identified interactions between dysferlin and over 100 other proteins, including many implicated in membrane repair [145,147,148]. Fluorescence-imaging experiments using mouse myoblasts show possible associations between dysferlin, TRIM72, and caveolin-3 (Cav3) that facilitates intracellular vesicle trafficking during acute damage membrane repair [82]. Furthermore, scrape injury experiments show that TRIM72 colocalizes and accumulates with dysferlin at the injury site, forming a lattice-like network near the lesion edges [68,82]. Substitutions in Cav3 influence dysferlin trafficking with TRIM72, in which significant amounts of TRIM72 and dysferlin are redistributed to the Golgi apparatus suggesting functional interactions between TRIM72 and dysferlin that is mediated by Cav3 [82,149]. Substitutions in Cav3 or TRIM72 knockout experiments display abnormal vesicular trafficking in mouse myoblasts, whereas dysferlin null experiments show normal vesicle accumulation near the sarcolemma, but defective membrane repair [76,82]. This suggests that dysferlin participates in the resealing process that requires protein partners to facilitate vesicle recruitment to injury sites [82]. Supporting this idea, pulldown assays show association between the dysferlin C2A domain and TRIM72 in the absence and presence of Ca^2+^-free environment, that is dependent on the TRIM72 oligomerization state in an oxidation-dependent manner [150]. Substitutions in the dysferlin domain (W52R and V67D) that are causative for muscle disease also alter its Ca^2+^ association with TRIM72 [150].

Amongst the many dysferlin partners is the giant muscle-cell protein AHNAK, shown to migrate to a membrane injury site under cellular stress. AHHAK is a cytosolic protein that associates with vesicles (enlargosomes) involved in exocytosis [151] in a Ca^2+^-dependent manner. The absence of a transmembrane domain in AHNAK indicates its localization to a membrane surface is controlled by interactions with other partner proteins. Immunostaining of skeletal muscle shows colocalization of dysferlin and AHNAK at the sarcolemma of skeletal muscle [146]. Furthermore, coimmunoprecipitation coupled to mass spectrometry experiments shows interactions between dysferlin and AHNAK [146]. GST pulldown experiments indicate that the dysferlin C2A domain is most important for the recruitment of AHNAK [146]. This interaction appears to be calcium insensitive and dependent on the C-terminus of AHNAK. Proteomic experiments also identify the EF-hand protein S100A10 and phospholipid-binding protein ANX2 as members of the dysferlin complex [145,147]. Dysferlin and ANX2 co-accumulate at injury sites of the plasma membrane in a Ca^2+^-sensitive fashion [152].

ANX2 forms a tight complex with S100A10 and the two proteins are frequently coisolated as a S100A10:ANX2 heterotetramer [153,154]. Yeast three hybrid experiments show S100A10:ANX2 interacts with a C-terminal region from AHNAK different from that used by dysferlin [155]. Furthermore, structural experiments show that AHNAK asymmetrically spans the complete heterotetramer utilizing two both S100A10 and ANX2 protomers [156–158]. These interactions paint a picture where AHNAK acts as a scaffold to recruit both S100A10:ANX2 and dysferlin [146,156]. However, the precise arrangement of these proteins, along with other candidate repair proteins such as TRIM72 and Cav-3, is unknown as are the mechanisms used for the repair process.

### Cysteine protease calpain enzymes sense membrane ruptures

Calpains are a large family of Ca^2+^-sensitive cysteine proteases ubiquitously expressed as inactive proenzymes in most cell types [159]. Rather than being a degradative protease, calpains modify their substrate by cleavage and often confer a Ca^2+^-signaling function. Two of the best studied enzymes, calpain-1 and calpain-2 are composed of a large 80 kDa catalytic subunit (CAPN1, CAPN2) with two protease core domains (PC1, PC2) that bind two Ca^2+^ ions and a small 30 kDa noncatalytic subunit (CAPNS1) [160–163]. Each subunit also contains a penta EF-hand domain that binds four additional Ca^2+^ ions. The cleavage activities of calpains are closely correlated to extracellular Ca^2+^ levels and typically require high Ca^2+^ levels such as those that occur during plasma membrane rupture. For example, calpain-1 and calpain-2 require between 5–50 μM or 200–800 μM Ca^2+^ concentrations, respectively, to reach half-maximum activity [164]. Calpain-3 deficiency results in LGMD-recessive type 1 (LGMDR1) but has little effect on the survival of laser-wounded skeletal myotubes [165]. Decreased expression of calpain-1 and calpain-2 in mouse embryonic fibroblasts shows loss of sarcolemma-resealing activity, suggesting calpain-1 and -2 are required for calcium-dependent membrane repair [165]. Moreover, knockout of CAPNS1 inhibits Ca^2+^-dependent resealing of membrane ruptures. However, knockout of either calpain-1 or calpain-2 has less effect on membrane repair, suggesting redundancy for protein cleavage events during membrane repair [165]. Consistent with a role in the repair of larger lesions, loss of both calpains decreases cell survival following mechanical membrane injury but is not impacted by small ruptures formed by SLO and saponin [166]. High-resolution microscopy shows that calpain inhibition prevents resealing of crayfish medial giant axons but does not prevent vesicle accumulation [167]. These findings indicate that calpains are required for Ca^2+^-dependent survival after membrane injury [164] likely due to enhancing membrane fusion rather than vesical formation and protein recruitment at the injury site [167].

Calpains-1 and -2 proteolyze dysferlin between the C2E and C2F domains yielding a membrane bound C-terminal fragment [168]. Proteolysis requires extracellular Ca^2+^ provided by membrane rupture [68]. Interestingly, a genetic deletion in the dysferlin gene has also been identified that yields a similar C-terminal protein fragment (‘mini-dysferlin’; includes C2F, C2G, and transmembrane domains) [169]. This dysferlin fragment resembles inverted Syt1, having its transmembrane anchor at the C-terminus rather than the N-terminus. When truncated dysferlin is expressed in dysferlin-null mice, isolated mice muscle cells show restored membrane repair function [169]. Furthermore, immunolabeling experiments show that the C-terminal fragment of dysferlin is detected at sites of membrane injury in human myoblasts, rather than the N-terminus [68,168]. Other experiments reveal that TRIM72 and mini-dysferlin-containing vesicles cluster at injury sites within 10 s following injury [68,170]. Mini-dysferlin and TRIM72 aggregate with exposed phospholipids to encircle the injury site, thus stabilizing the plasma membrane [68]. This mechanism appears to be most prominent in lung, liver, pancreas, and placenta (40–60%) rather than cardiac or skeletal muscle cells (10–15%) where low abundance the mini-dysferlin protein is detected [168]. Calpain-3 is also proposed to work as a regulatory enzyme for the dysferlin complex [171]. Immunostaining in cell cultures suggests cleavage of AHNAK and the muscle specific-protein titin is regulated by calpain-3 perhaps, suggesting a role following membrane repair linked to cytoskeletal maintenance [166,172].

### Annexin complexes seal a membrane

The annexins are a family of Ca^2+^-regulated phospholipid-binding proteins, with Annexins A1, A2, A4, A5, and A6 most known for their roles in membrane repair. Annexins include four to eight core domains that bind Ca^2+^ arranged in a right-handed superhelix and a unique, variable length N-terminal domain [173]. Under resting Ca^2+^ concentrations, most annexins diffusely distribute in their Ca^2+^-free, inactivated state in the cytosol. In response to a Ca^2+^ influx from membrane injury, Ca^2+^ binding to the outer surface of the annexin protein facilitates interaction with the acidic phospholipid membrane. Calcium-binding sites in the annexins are located in the outer loop regions of the protein and display different calcium co-ordination patterns from EF-hand calcium-binding motifs [174,175]. Although the core domains are highly conserved in the annexin family, their relative orientations and numbers of bound Ca^2+^ ions differ amongst isoforms [175]. Crystal structures of Annexins A1 and A5 show six and four Ca^2+^-binding sites, respectively [176,178]. It is thought that the range of calcium sensitivities by the annexins may be correlated with the size of the membrane hole. Furthermore, Ca^2+^ sensitivities for individual annexin proteins vary in the presence of phospholipids. For example, ANX1 and ANX2 have calcium *K*_d_ values of >1000 and 100–500 μM, respectively, without phospholipid [179–181,228]. In contrast, their affinities for Ca^2+^ increase in 10- to 100-fold with phospholipid present [177,179,228,229]. The higher calcium sensitivity of ANX2 may be due to its association with S100A10 that is unable to bind calcium (see below) [182]. The variable N-terminal domains of annexins are thought to provide specificity for different binding partners and in most cases these interactions are controlled by Ca^2+^ binding to the annexin protein. For instance, the N-terminal domain of ANX1 is buried within the core domains in the absence of Ca^2+^ and is released upon Ca^2+^ binding due to rearrangement of the core domains [183].

The relatively weak affinities and preference for phospholipid membranes make members of the annexin family perfect candidates for membrane repair. Furthermore, the variable N-termini provides a platform for binding partners found in different membrane-resealing pathways. Frequent binding partners for the annexin proteins are the S100 proteins, a family of dimeric, EF-hand proteins. For instance, the ANX1 and ANX2 co-ordinate with S100A11 and S100A10, respectively [157,158,184,185]. Most interactions of annexins with S100 proteins are also controlled by Ca^2+^ binding to the S100 protein, which typically bind two Ca2+ ions per protomer and have Ca^2+^ dissociation constants ranging from 10 to 500 μM for the two Ca^2+^-binding sites [186–188]. Calcium binding to most S100 proteins causes a significant conformational change that provides a hydrophobic surface used to recruit the N-terminal region from an annexin protein [184]. Numerous three-dimensional structures show the annexin N-terminus forms an amphipathic helix that bridges the S100 dimer [184,185,188]. Although annexins are found as monomers in the cytosol, binding to a dimeric S100 protein results in the formation of a noncovalent heterotetramer. One outlier in this mechanism is S100A10 that recruits ANX2 in the absence of Ca^2+^. S100A10 is a unique member of the S100 family that has lost its ability to bind Ca^2+^ yet retains the Ca^2+^-activated structure exhibited by other Ca^2+^-bound S100 proteins [154,190]. The S100A10:ANX2 complex was originally isolated as a Ca^2+^-insensitive p11p36 heterotetramer from intestinal epithelium cells [154]. Although other S100 proteins such as S100A4 and S100A11 can recruit ANX2 [191–195], S100A10 has been shown to bind to this annexin protein nearly three-orders of magnitude stronger. The S100A10:ANX2 complex is likely the predominant form used in membrane repair. In the absence of ANX2, S100A10 is rapidly degraded in cells [196]. In addition, it has been observed that oxidative stress results in glutathionylation of ANX2 in the S100A10:ANX2 heterotetramer, resulting in a loss of its ability to interact with phospholipids and F-actin, suggesting ANX2 is sensitive to oxidation that alters its conformation and function [197].

ANX1 and ANX2 aggregate with intracellular vesicles by interacting with dysferlin in a Ca^2+^-dependent manner. Both ANX1 and ANX2 colocalize with dysferlin at the sarcolemma [53]. This association is disrupted in muscle cells lacking dysferlin [53], suggesting an interaction between dysferlin and annexins may be essential for vesical aggregation and fusion during membrane repair. ANX2 is known to stabilize and organize lipid microdomains and is proposed to regulate exocytosis events in chromaffin cells [198]. The S100A10:ANX2 complex also localizes at membrane injury sites [156]. Both ANX1 and ANX2 translocate to the membrane in damaged human and zebrafish muscle cells, although at different rates that may depend on localization prior to a rupture [199,200]. Annexin knockout experiments display poor myofiber repair or muscle regeneration abilities [201–203]. A Ca^2+^-insensitive version of ANX1 also inhibits membrane sealing [204]. Association between dysferlin and the S100A10:ANX2 complex is Ca^2+^ and injury dependent. Furthermore, a proposed model depicts that membrane-patching repair requires the interaction between dysferlin and ANX1 and ANX2 [205]. Abnormal localization of ANX1 and ANX2 has been found in dysferlin-deficient mouse muscle cells [53]. Interestingly, fluorescence lifetime imaging microscopy (FLIM) experiments demonstrate significant interactions between dysferlin and ANX1 during resting physiological Ca^2+^ levels, and this interaction is lost in injured cells [53]. On the other hand, interactions between dysferlin and ANX2 are observed in resting and ruptured mice muscle cells, suggesting that each annexin protein may have a specific role in repair processes [53,206]. The recruitment of Ca^2+^-activated ANX1 to a damaged region seems independent of S100A11. Immunolocalization assays show ANX1 concentrates around the rupture site in human breast cancer cells lacking S100A11 expression [205]. Interestingly, ANX1-deficient mice show little effect in Ca^2+^-dependent myofiber repair compared with wild-type myofibers. Instead, increasing numbers of unfused differentiating myoblast suggest that the cell–cell fusion stage of myofiber formation was affected by the absence of ANX1, implying that ANX1 potentially participates in regulating membrane fusion, and other possible proteins may substitute its role in membrane repair [201].

Other annexin proteins are proposed to be important in membrane repair. Annexin A5 (ANX5) is rapidly recruited to a membrane injury site and binds to the curved edges of a damaged membrane. ANX5 self-assembles into two-dimensional (2D) protein arrays on PS-containing membranes, possibly stabilizing the membrane, and limiting wound expansion during membrane repair [207–209]. ANX5-null cells exhibit defective membrane repair that is reversed upon addition of recombinant ANX5 [210]. In contrast, an ANX5 variant that is unable to assemble into arrays, fails to rescue membrane repair ability.

Annexin A4 (ANX4) and A6 (ANX6) appear to work together to induce membrane curvature and wound closure. The smallest annexin member, ANX4, self-assembles into trimers on a membrane surface to induce curvature at rupture edges [211,212], allowing ANX6 to constrict and close of the wound [212]. Truncated ANX6 competes with and prevents translocation of wild-type ANX6 to alter assembly of the annexin repair complex [213]. Conversely, overexpression of ANX6 promotes membrane repair by increasing the formation of external vesicles at the site of membrane injury [213]. This repair strategy seems to depend on cell type and wound size, in which smaller lesions in metastatic cancer cells require actin remodeling and membrane restoration by the S100A10:ANX2 complex. On the other hand, larger wounds in metastatic cancer cells involve S100A10:ANX2 coupled to ANX4 and ANX6 for repair, suggesting diverse membrane repair mechanisms may be used to maintain membrane integrity [212].

### ALG-2 and ESCRT-mediated repair

ALG-2 (also PDCD6) is a highly conserved Ca^2+^-binding protein in the penta-EF-hand (PEF) family. Although originally thought to be a proapoptotic signaling protein, ALG-2-deficient mice display little phenotypic changes to support this function [214]. Instead, ALG-2 displays an array of interacting protein partners that support a role in cell proliferation, vesicle transport, and membrane repair in many cell types [215–217]. ALG-2 includes a PEF domain characterized by utilizing the fifth EF hand for Ca^2+^-independent dimer formation [218,219]. The first (EF-1) and third (EF-3) EF hands in ALG-2 possess the two highest-affinity Ca^2+^-binding sites with *K*_d_ of 1.2 and 300 μM, while EF-5 displays very weak Ca^2+^ binding. Ca^2+^ binding to EF-1 and EF-3 induces conformational changes that open a hydrophobic pocket near the N-terminus of ALG-2 for target recognition and binding, suggesting ALG-2 is a calcium sensor [218,219]. As a result, substitutions to the Ca^2+^ co-ordinating residues in EF-1 and EF-3 impairs Ca^2+^-induced conformational changes that may directly alter ALG-2 function [220]. Consistent with this, immunofluorescence assays show ALG-2 localizes to the cytoplasm and nucleus of cells [221] but moves to the membrane fraction in the presence of Ca^2+^ [222].

**Figure 5:**
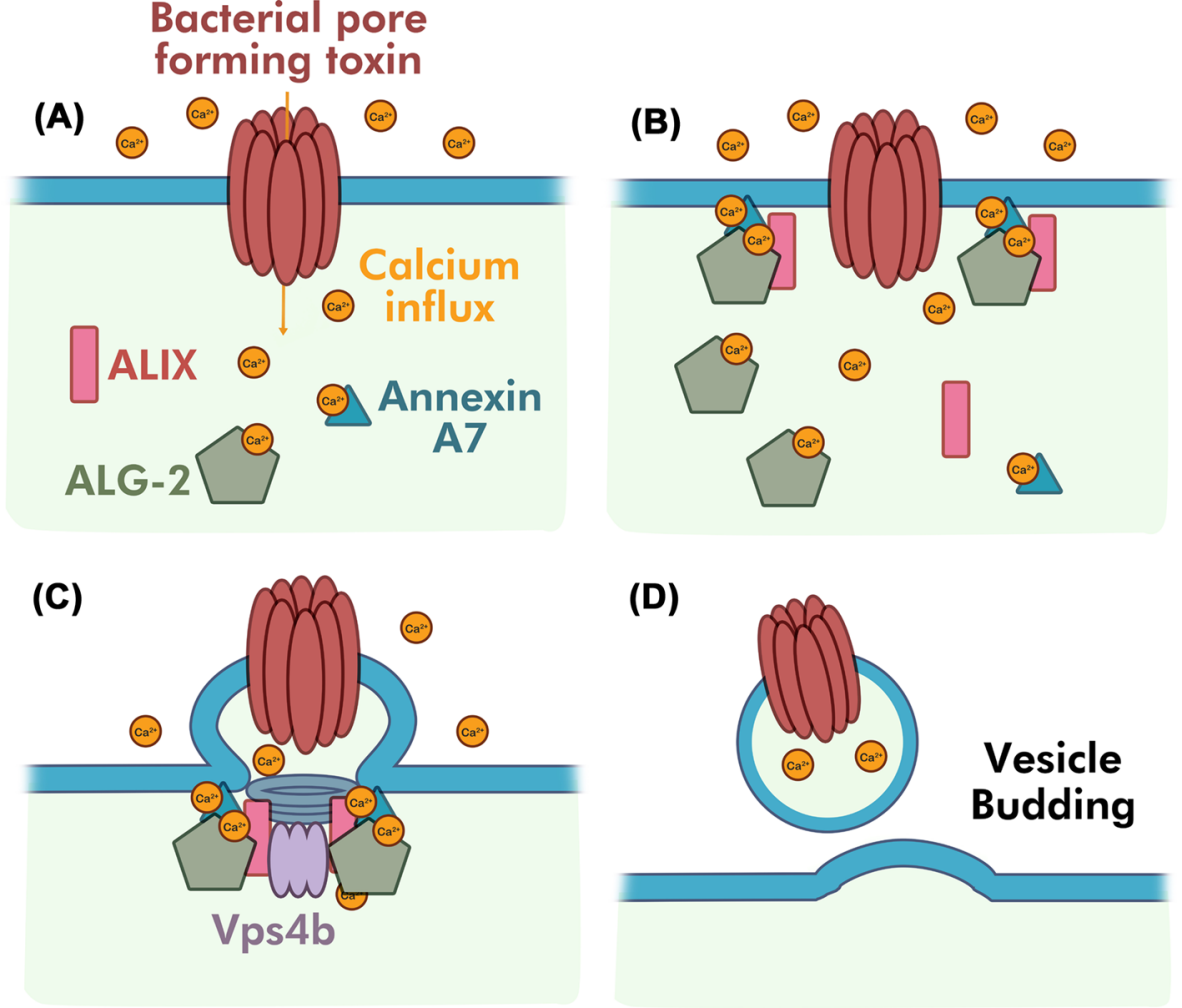
Externalization repair via ESCRT-mediated shedding to remove bacterial PFTs (**A**) PFTs induce stable transmembrane protein-based lesions, which cannot simply repair by spontaneous membrane reseal. Ca^2+^ activates ANX7 and PEF protein ALG-2. ANX7 associate with ALG-2, and Ca^2+^-activated ALG-2 associate with ALIX. (**B**) ANX7, ALG-2, and ALIX form a complex that migrates to the plasma membrane. (**C**) ALG-2 complex initiates the assembly of ESCRT-III and Vps4 at the membrane injury. (**D**) Sequential membrane remodeling and membrane scission by ESCRT pathway. Other ESCRT distinct complexes involve in the recruitment and membrane repair process are not shown here.

A variety of ALG-2-interacting proteins have been identified that associate with vesicle and plasma membranes, including Annexins A7 (ANX7) and A11, ALG-2-interacting protein X (ALIX), and components of the ESCRT complex [218,227]. Membrane-wounding assays show Ca^2+^-bound ANX7 nucleates at ruptured membrane edges along with ALG-2 following scrape injury, suggesting that ANX7 regulates the proper localization of ALG-2 to the injured membrane [227]. ESCRT proteins are organized into five functionally distinct complexes, shown to facilitate the repair of narrow membrane wounds (<100 nm) observed in PFT-injured cells [71]. Laser-induced membrane damage experiments show that ESCRTs facilitate cleavage of damaged regions of the cell membrane using ALG-2 and ALIX [223]. Particularly, ESCRT-III induces membrane deformation and scission to shed extracellular vesicles to remove damaged membranes [71,223,224]. The essential regulator protein ALIX has an important role in the recruitment of ESCRT-III components including CHMP4 [216,223,225,226]. On the other hand, knockdown of ALG-2 inhibits the recruitment of ALIX at the injury site but not vice versa [223]. Thus, Scheffer et al. proposed a model whereby ESCRT-mediated membrane repair requires the use of Ca^2+^-activated ALG-2 to initiate the sequential recruitment of ALIX and ESCRT proteins to the lesion for membrane repair (Figure 5) [223,228].

Recent studies used cell viability following electroporation membrane lesions as a measure for membrane repair. An ALG-2 variant lacking the ability to bind Ca^2+^ due to substitutions in the EF-1 and EF-3 domains shows lower cell survival compared with controls [217]. On the other hand, overexpression of wild-type ALG-2 increases cell viability of lesion-damaged cells [217]. Furthermore, cell viability decreases when the ALIX-binding site on ALG-2 is blocked, supporting the importance of an ALG-2/ALIX interaction for the repair model proposed by Scheffer [217,223].

## Conclusions

Plasma membrane repair is an emergency response system, essential for maintaining cellular homeostasis and ensuring cell survival. The rapid influx of extracellular Ca^2+^ upon membrane rupture is one of the primary signals to initiate the repair process. Recruitment of multiple Ca^2+^-dependent sensor proteins has been identified that display multiple membrane repair mechanisms. It is likely that these protein sensors co-ordinate a diverse set of repair responses tailored to the location, size, and nature of the membrane injury. Defective membrane repair, frequently due to mutations in genes that code for repair proteins, is now recognized as a contributor to different forms of MD muscle disease and heart disease. Considering all known protein components work together and have distinct roles in membrane repair it is likely that no single mechanism, nor therapeutic solution, applies in all injury scenarios. Although some three-dimensional structure information is available for some individual proteins the atomic level details of the interactions between nearly all repair proteins are missing. This information is urgently needed as the foundation for possible pharmaceutical therapies. In addition, it is likely that other possible mediators and physiological conditions exist that control membrane repair mechanisms that have yet to be resolved. Many of these aspects should be identified to gain a full understanding of the membrane repair machinery and provide new avenues for future treatments of inherited diseases.

## Abbreviations

2D: two-dimensional
ALG-2: apoptosis-linked gene 2
ALIX: ALG-2-interacting protein X
ANX1: annexin A1
ANX2: annexin A2
ANX4: annexin A4
ANX5: annexin A5
ANX6: annexin A6
Ca2+: calcium
CRISPR: clustered regularly interspaced short palindromic repeats
ESCRT: endosomal sorting complex required for transport
FLIM: fluorescence lifetime imaging microscopy
Kd: dissociation constant
LGMD2B: limb-girdle muscular dystrophy type 2B
LGMDR1: limb-girdle muscular dystrophy recessive type 1
MD: muscular dystrophy
PEF: penta-EF-hand
PFT: pore-forming toxin
PS: phosphatidylserine
SNAP-25: 25 kDa synaptosomal-associated protein
SNARE: Soluble N-ethylmaleimide sensitive factor Attachment protein Receptor
Syt: synaptotagmin
Syt7: synaptotagmin VII
